# The Environment as a Patient: A Content Analysis of Canadian Nursing Organizations and Regulatory Bodies Policies on Environmental Health

**DOI:** 10.1177/08445621211035913

**Published:** 2021-10-20

**Authors:** Courtney Mundie, Lorie Donelle

**Affiliations:** 1 Arthur Labatt Family School of Nursing, 6221 Western University, London, Ontario, Canada

**Keywords:** nursing, environmental health, climate change, policy analysis

## Abstract

**Background:**

Individual, community, and societal health is impacted by the environment, specifically by air, water and soil pollution, and climate change. Poor environmental conditions have been associated with many illness exacerbations. Although global nursing organizations have increased their environmental health focus, evidence is lacking that Canadian nurse leaders and organizations are similarly invested.

**Purpose:**

The purpose of this analysis was to explore the policies of Canadian nursing regulatory bodies and associations on nursing practice specific to environmental health.

**Methods:**

A content analysis of nursing focused position statements and competency documents was conducted to assess Canadian nursing policies in environmental health. Publicly available position statements and competency documents regarding health and the environment were retrieved from Canadian nursing regulatory colleges and nursing associations, the Canadian Nursing Associations, and the International Council of Nurses. All documents were coded inductively and thematically analyzed.

**Results:**

In total, 22 documents were retrieved which consisted of 11 policy statements from nursing associations and 11 competency documents from nursing regulators and national associations. Four themes were generated: collaboration, language of engagement, nursing actions, and social justice.

**Conclusion:**

There is a gap between nursing policies and competencies directing nursing action related to the health of the environment across Canada. There is an opportunity to improve eco-literacy within the nursing profession, undergraduate education and to produce nursing research on environmental health.

## Background and Purpose

The World Health Organization created the Commission of Social Health Determinants in 2005 to outline actions for enhanced health and to mitigate health inequities across the globe (Baum, 2018; World Health Organization, 2020). The report generated by the Commission affirmed that improving daily living situations, reducing wealth and resource inequities, and monitoring global health inequities are three key actions needed to improve global health (WHO Commission on Social Determinants of Health, 2008). Of the 17 sustainable development goals created, seven are directly related to environmental health: climate change, affordable and clean energy, sustainable cities and communities, responsible consumption and production, clean water and sanitation, life below water, and life on land (Lilienfeld et al., 2018; United Nations, 2020).

Environmental health is defined as promoting human well-being through limiting exposure to hazardous agents and conditions in the environment around a person, such as water, soil, air, and food pollution (Jackman-Murphy, 2015; Kalogirou et al., 2020; National Environmental Health Association, 2020). Poor environmental health is linked to poor human health, with a higher burden of these effects felt in low and middle-income countries (Frumkin & Haines, 2019; Nicholas & Breakey, 2017). Hazardous agents in the physical environment, as in air pollution, have been identified as a contributing factor to a variety of illnesses, including ischemic heart disease, chronic obstructive pulmonary disorder and cancer, and thus, is linked to ∼4.2 million deaths worldwide every year (Frumkin & Haines, 2019). Although the effects of poor environmental health are well documented (Frumkin & Haines, 2019; Goodman, 2013; World Health Organization, 2018), advocacy efforts in the health care professions to improve environmental health have received little attention (Lilienfeld et al., 2018).

Despite the advances of the profession and nursing science within the last 3 decades, the stereotypical nursing role is of a direct care provider at the bedside working with patients (ten Hoeve et al., 2014). The professional opportunities available to nurses have significantly expanded, and nurses are present within leadership, academia, research, clinical, and government roles (ten Hoeve et al., 2014). Nurses who practice from determinants of health framework acknowledge a diversity of “patients,” inclusive of individuals, families, communities, global populations, and increasingly, the environment.

Similarly, nurses have a diverse practice portfolio, inclusive of direct patient care, research, policy development, health advocacy and health activism. An activist is someone who tries to draw public attention and concern to an issue they consider to be important (Parsons, 2016), and “who works to bring about political or social changes by campaigning in public or working for an organization” (Collins, 2020, para. 1). Activism can play a vital role in society through raising awareness of important issues, such as environmental threats or civil rights issues. Nursing activism moves nursing practice from a passive role to one of taking action to influence change (Florell, 2020). Florell (2020) contends that nursing practice is historically rooted in social and political activism and, within contemporary nursing, activism practices are diminished and undervalued (Florell, 2020). However, health activism is an emerging but crucial nursing role that utilizes caring and compassionate action to alleviate health inequities and social injustice for communities and global populations and is critical to improving the environmental determinants of health (Florell, 2020).

The International Council of Nurses [ICN] (2019) calls on all nurses to advocate for environmental health policy and climate change mitigation. Global change requires a multitude of actors to champion policy change and legislation addressing social, health, and environmental inequities (Aranda, 2019; MacDonnell & Buck-McFadyen, 2016). Therefore, it is important for nurses around the globe, including Canada, to create a political presence in environmental health (ICN, 2019; Lilienfeld et al., 2018).

## Nurses Role in Environmental Health

Researchers have reported that poor environmental health leads to a host of human illnesses, such as increased rates of vector-borne illnesses, malnutrition, respiratory and cardiac illnesses, and cancer (Lilienfeld et al., 2018; Nicholas & Breakey, 2017). Environmental health is increasingly recognized as an important focus of nursing practice, and researchers have issued a call for nurses to incorporate environmental health leadership, advocacy, and activism roles as part of nursing practice (Goodman, 2013, 2015; Leffers & Butterfield, 2018; Lilienfeld et al., 2018; Nicholas et al., 2020; Nicholas & Breakey, 2017). Florell urges nurses to consider activism as a way to re-focus professional accountability for the multiple and intersecting factors that determine health and to extend nursing practice beyond the bedside to take action on the environmental, socioeconomic, and sociopolitical determinants of health. This will require a renewed focus on public and environmental health activism. Polivka et al. (2012) found that American nurses had minimal knowledge and skills regarding the health effects of climate change, suggesting nurses are currently ill-prepared to recognize and protect environmental health.

Furthermore, existing nursing curriculums lack an environmental health focus and researchers have called for an increase of eco-literacy (awareness of environmental concerns and the knowledge of how to prevent them) through education (Larsson & Butterfield, 2002; Leffers & Butterfield, 2018; Neal-Boylan et al., 2019; Polivka & Chaudry, 2018; Potter, 2019; Sayre et al., 2010; Valentine-Maher et al., 2018). Researchers agree that the next generation of nurses will lack the knowledge and skills concerning the complex interactions between environmental health and human health and without educational intervention, their capacity to lead transformative change to reduce the environmental effects on human health will be hindered.

Currently, economic restrictions, lack of time and lack of intradisciplinary support are key barriers for nurses engaging in environmental health advocacy (Buck-McFadyen & MacDonnell, 2017; Nicholas & Breakey, 2019; Terry & Bowman, 2020). Nurses perceive health care organizations and facilities as predominantly focused on fiscal accountability, which discourages professionals from advocating for organizations to switch to renewable energy sources or low waste equipment options due to higher upfront costs (Nicholas & Breakey, 2017). Terry and Bowman's (2020) study of environmental health nursing activists found a troubling trend where nurses felt ostracized from their colleagues when discussing their environmental views in the workplace, leading to more personal action than professional. Evidence suggests that the lack of professional support in this regard creates high emotional burnout for nurses and reduces their motivation to advocate in a professional context (Terry & Bowman, 2020). Greater environmental health awareness and support from nursing colleagues is required to empower activists in the fight for environmental health (Terry & Bowman, 2020).

In the United States, the nursing profession has responded to the call for environmental health advocacy with the establishment of the Alliance of Nurses for Healthy Environments (ANHE), founded in 2008, to collaborate efforts across the United States in advancing environmental health nursing knowledge (Leffers et al., 2014). Leffers et al. (2017) discussed the growth of the environmental health nursing role in the United States as a result of the ANHE, and how nursing education in environmental health is now included in nursing curriculums across the country and recognized as a nursing specialization. Other countries with an environmental health nursing research focus include Sweden (Anåker et al., 2015) and the United Kingdom (Terry & Bowman, 2020).

There is little research evidence to indicate the Canadian nursing profession is actively engaged in practice focused on the health of the environment. There were no articles that investigated environmental health nursing activism within the Canadian context. MacDonnell and Buck-McFadyen (2016) found nurses who engaged in political activism have experienced numerous consequences, such as job loss and ostracization from organizations and communities. With an increase of policies mandating how nurses can engage with the media, participants found that their ability to be politically active was constrained (MacDonnell & Buck-McFadyen, 2016). The subsequent 2017 study by Buck-McFadyen and MacDonnell echoed this sentiment and reported that the practice of nursing activism in Ontario was affected by how nurse educators understood and encouraged activism within the curriculums. The lack of Canadian based research on nursing activism for environmental health may be related to a lack of environmental health focus in current Canadian nursing practice. Hanley and Jakubec’s (2019) study confirmed the lack of environmental health focus for Canadian nurses, and their study participants stated environmental health concepts should be incorporated into the undergraduate curriculum to address the knowledge-practice gap.

Regulatory colleges in Canada are mandated to protect the public through establishing requirements for entry to practice, enforcing practice standards and ensuring members are continuing competence (Canadian Nurses Association, 2021; College of Nurses of Ontario, 2020b). Competency documents are also used to evaluate nursing programs to ensure new graduates achieve practice standards (College of Nurses of Ontario, 2021). In addition, professional nursing associations help create and disseminate evidence-based practice documents focused on nursing competencies (Holleman et al., 2006). Nursing colleges and associations influence nursing education and practice. Therefore, the purpose of this research was to explore the policies of Canadian nursing regulatory bodies and associations focusing on nursing practice specific to environmental health.

## Methods

A content analysis was used to explore the role of Canadian nursing leadership regarding nursing action and care of environmental health. The aim of this content analysis was to assess the publicly available policies and position statements published by provincial and territorial nursing regulatory bodies and Canadian and international nursing associations. The International Council of Nurses was included in this content analysis as the council's mission is to represent nurses worldwide, as well as advocate for health in all policies (International Council of Nurses, 2021). Therefore, their work affects the organizations and associations within Canada. This content analysis follows the steps outlined by Smith (2000), complimented by the framework discussed by Elo and Kyngäs (2008). The research question guiding this analysis is “How do the policies of International, and Canadian nursing regulatory bodies and associations reflect nursing practice focused on environmental health?”

### Data and Methods

A search of provincial, territorial, and national nursing regulatory bodies and associations across Canada, as well as the Canadian Nurses' Association (CNA) and International Council of Nurses (ICN), was conducted. The websites’ policy and position statement section and search engines were utilized to find all documents related to “environmental,” “environmental health,” “climate,” and “climate change.” The search included all documents published online up to November 2020. Documents were included in the analysis if they were written in English or French, and were identified as position statements, policies or competency documents. Google Translate was used for French to English translation for French language documents. Google Translate is ∼75% accurate in translating Western European languages (Balk et al., 2013; Patil & Davies, 2014). The Google Translation result was assessed and confirmed by a third-party bilingual individual. The documents retrieved included policies, position statements and registered nurses’ competency documents from colleges and associations, as these documents indicate an organization's stance on a subject and direct nursing practice (Hewison, 2007). Webinars, conference notes and letters were excluded from retrieval, as these documents are often subjective, derived from a formal policy or position statement, and represent microlevel interventions that do not set direction for an organization. Published materials from nursing unions across Canada were also excluded as unions were created to build worker power and stand up for safe workplaces, and therefore did not influence or direct nursing practice in public health (Canadian Labour Congress, 2019).

### Data Analyses

An inductive coding process was used for this content analysis (Elo & Kyngäs, 2008). Each document was read thoroughly by the primary coder. Three documents were coded independently by two analysts, and the subsequent codes were reviewed, categorized, and amalgamated to create the final coding system, which was applied by the primary analyst to each document using NVivo 12 (2018). Themes were identified from the coding nodes and further analyzed.

### Findings

#### Descriptive Findings

A total of 22 documents were retrieved for this analysis (see Table 1). There were ten nursing practice competency statements retrieved; a single competency document was retrieved from 10 of the 12 provincial and territorial nursing regulatory colleges. Competency documents were not found through the online search from the Yukon Registered Nurses’ Association and the Ordre des infirmières et infirmiers du Québec (OIIQ). The CNA Code of Ethics document was referenced in most of the competency documents retrieved, and therefore was also included in the analysis. The remaining 11 documents were position statements from four provinces, the CNA and ICN (as shown in Table 1). Six provincial and the two territorial nursing organizations (66% of the Canadian nursing organizations) did not have publicly available position statements outlining their stance regarding nursing practice and environmental health. The CNA and Canadian Medical Association (CMA) collaborated on one position statement, which was included as one of the five statements published by the CNA. Five position statements were published between 2000 and 2010, five were published between 2011 and November 2020, and one position statement lacked a date (as shown in Table 2). No position statements were retrieved from nursing associations in Atlantic Canada or the northern Territories (as shown in Table 1). All competency documents were published between 2010 and 2020 (as shown in Table 2).

**Table 1.** A list detailing which provinces had colleges and associations publish documents pertaining to environmental health.

Province	Position statements/policies published	Competency documents
British Columbia	1	1
Alberta	1	1
Saskatchewan	0	1
Manitoba	0	1
Ontario	2	1
Quebec	1	0
New Brunswick	0	1
Nova Scotia	0	1
Prince Edward Island	0	1
Newfoundland and Labrador	0	1
Nunavut and Northwest Territories	0	1
Yukon	0	0
Canada (CNA)	5	Code of Ethics
ICN	1	

**Table 2.** A list detailing which documents were retrieved from each association or college for this analysis.

Position statement or competency document	Year	Location; organization
Global health and equity	2009	Canada; CNA
Joint position statement: Toward an environmentally responsible Canadian health sector	2009	Canada; CNA
Joint position statement: Environmentally responsible activity in the health-care sector	2009	Canada; CNA
Nurses and environmental health	2017	Canada; CNA
Climate change and health	2017	Canada; CNA
Code of ethics	2017	Canada; CNA and CMA
Position statement on vulnerability	2005	Alberta; CARNA
ELCs for the practice of registered nurses	2019	Alberta; CARNA
Engaging BC nurses with climate change issues	No date	BC; NNPBC
Competencies in the context of entry level registered nurse practice in BC	2015	BC; CRNBC
Registered nurse entry-level competencies	2019	Saskatchewan; SRNA
ELCs for the practice of registered nurses	2019	Manitoba; CRNM
Entry-to-practice competencies for registered nurses	2020	Ontario; CNO
Environmental carcinogens and health	2008	Ontario; RNAO
Healthy energy solutions for Ontario	2011	Ontario; RNAO
Les impacts des changements climatiques sur la santé des populations et la pratique infirmière	2019	Quebec; OIIQ
ELCs for the practice of registered nurses in New Brunswick	2020	New Brunswick; NANB
ELCs for the practice of registered nurses	2020	Nova Scotia; NSCN
ELCs for registered nurses	2019	Prince Edward Island; CRNPEI
ELCs for the practice of registered nurses	2019	Newfoundland and Labrador; ARNNL
ELCs for the practice of registered nurses	2019	Northwest Territories and Nunavut; RNANT/NU
Nurses, climate change and health	2018	International; ICN

ARNNL = Association of Registered Nurses of Newfoundland and Labrador; BC = British Columbia; CNA = Canadian Nurses' Association; CMA = Canadian Medical Association; CNO = College of Nurses of Ontario; CARNA = College and Association of Registered Nurses of Alberta; CRNBC=College of Registered Nurses of British Columbia; CRNPEI = College Of Registered Nurses Of Prince Edward Island; ELC = entry-level competencies; ICN = International Council of Nurses; NANB = Nurses Association of New Brunswick; NNPDC = Nurses and Nurse Practitioners of British Columbia; SRNA = Saskatchewan Registered Nurses Association;RNAO = Registered Nurses Association of Ontario; RNANT/NU = Registered Nurses Association of Northwest Territories and Nunavut; OIIQ = Ordre des infirmières et infirmiers du Québec;

The policies varied between having environmental health as the main focus or including it in a brief acknowledgment. Eight position statements included environmental health as the main focus of the document, and discussed how the health of the environment is crucial to maintaining and improving human health (CNA, 2009b, 2017a, 2017b; CNA & CMA, 2009; ICN, 2018; Nurses and Nurse Practitioners of British Columbia [NNPBC], n.d.; Ordre des infirmières et infirmiers du Québec [OIIQ], 2019; Registered Nurses Association of Ontario [RNAO], 2011). The physical environment and climate is identified as a critical determinant for human health, and nurses are encouraged to consider environmental health and the effects of climate change as a priority in professional practice (CNA & CMA, 2009; ICN, 2018; NNPBC, n.d.; OIIQ, 2019; RNAO, 2011).

The remaining three position statements mentioned environmental health briefly within their documents. One of the position statements from the RNAO discussed poor environmental health as a contributor to cancer (RNAO, 2008) while two other position statements from the CNA and CARNA commented on the relationship between improved environmental health and the positive impact on social justice and health equity (CNA, 2009a; College and Association of Registered Nurses of Alberta [CARNA], 2005). The CNA (2009a, 2009b) noted that a stable ecosystem is listed as a prerequisite for health and a basic human right within the Ottawa Charter for Health Promotion.

#### Themes

Four themes were generated from the content analysis of the competency documents and position statements. The themes identified are as follows: (a) *Collaboration*, (b) *Language of Engagement*, (c) *Nursing Actions,* and (d) *Social Justice*.

### Collaboration

Nursing collaboration with a variety of key stakeholders was found to be a key element in promoting environmental health. The CNA (2017a, 2017b, 2017c) stated “… that intersectoral and interdisciplinary collaboration, within and outside the health system, are crucial to nurses’ work in environmental health” (p. 3). The ICN (2018) also stressed the importance of collaboration by stating that nurses need to “collaborate with other health professional organizations, intergovernmental organizations, environmental and health organizations and other civil society groups” (p. 4). Nursing collaboration across sectors and with other disciplines was reiterated within nine of the 11 position statements to meaningfully impact environmental health, nurses need to collaborate with other sectors, disciplines, and the public to implement actions, policies, and reduce greenhouse gas emissions (CNA, 2009a, 2009b, 2017a, 2017b; CNA & CMA, 2009; ICN, 2018; NNPBC, n.d.; RNAO, 2008, 2011)

The collaboration theme was also composed of two sub-themes Global Collaboration and Identified Partners. Nurse leaders identified global collaboration as necessary to reduce the impact of climate change on environmental health (CNA, 2009b; ICN, 2018; NNPBC, n.d.), and the Paris Accord (an international treaty for climate change that went into effect in 2016) was identified as crucial in unifying countries across the globe in the fight against climate change (CNA, 2017a, 2017b; ICN, 2018; United Nations Framework Convention on Climate Change, 2021). The RNAO (2008) noted that nursing organizations in the United Stated and Australia have advanced environmental health through the development of a targeted environmental health nursing role, which focuses on natural environment preservation.

The second sub-theme, identified partners, recognized which bodies were important for nurses to collaborate with to address environmental health. Seven of the 22 documents identified the Canadian government as a key partner, and noted that nurses must collaborate with all levels of government to incorporate aspects of environmental health into policy, research, and global action (CNA, 2009a, 2009b, 2017a, 2017b; ICN, 2018; NNPBC, n.d.; RNAO, 2011). Two provincial organizations highlighted indigenous populations as important collaborating partners to be included in policy decisions and climate change mitigation, as they have a unique spiritual and cultural connection to the land that is being affected by poor environmental health (NNPBC, n.d.; RNAO, 2011). Finally, nursing organizations across Canada, health organizations such as the Canadian Cancer Society and the Lung Association, and humanitarian organizations were all identified as key groups to collaborate together to reduce their own negative impact on the environment, raise awareness and encourage action regarding climate change and environmental health (CNA, 2009b, 2017b; ICN, 2018; NNPBC, n.d.; RNAO, 2008, 2011)

### Language of Engagement

.This content analysis identified that the rhetoric used within the 22 retrieved documents was important to determine if an association was active or passive in its encouragement of nurses to advocate for environmental health. Language of engagement was defined and coded as words and phrases used by the organization related to nurses’ participation in environmental health, and how it directed its’ members to engage in environmental health care. The language used in these position statements claims nursing, as a profession, should be leading the wave of environmentally healthy activities at all levels. “Nursing is well positioned to use its professional voice in a unified way” (NNPBC, n.d., 3) and “nurses need to need to promote climate change adaptation … and mitigation” (CNA, 2017a, 1). The language of these organizations’ statements claims that nurses can increase environmental health attention in micro and macro levels of organizations and governments.

Nine position statements possessed similar language of engagement. Nursing organizations described their activities as championing and supporting the need to address environmental health at different levels of government and encouraging nurses to adopt environmentally responsible behavior into their professional practice and personal lives (CNA, 2009b, 2017b, 2017a; CNA & CMA, 2009; ICN, 2018; NNPBC, n.d.; OIIQ, 2019; RNAO, 2008, 2011). However, these same organizations failed to include detailed strategies for nurses to incorporate key ecological and health determinants into their practice. The NNPBC (n.d.) supports “the need to address climate change at regional, provincial, and national levels” (p. 1) and for “individual nurses to practice in a manner that integrates both ecological and social determinants of health” (p. 3). The CNA and CMA (2009) echo this form of engagement by asserting in their joint position statement that health care professionals “should begin by setting the example of responsibility in their own personal and professional lives” (p. 5). These five organizations state that health care professionals and nurses are called upon, encouraged and have a responsibility to advocate for environmentally sustainable practices, legislation and lifestyle choices (CNA, 2017b; CNA & Canadian Medical Association, 2009; NNPBC, n.d.).

### Nursing Actions

This theme was defined as the practice and action recommendations made by each organization in their published position statements and consists of four sub-themes: general practice recommendations, macro recommendations, pragmatic practice recommendations, and research recommendations.

General practice recommendations consisted of recommendations made by the nursing leaders that encourage nursing action in ambiguous terminology. Examples of this sub-theme include “nurses are called upon to educate themselves” (NNPBC, n.d., 4), “Canadian health professionals … have the right and responsibility to … participate in finding solutions” (CNA, 2009a, 1) and nurses should “(raise) awareness of clients and the public” (RNAO, 2008, 4). Regulatory nursing bodies across the country encourage general nursing action as well, stating that nurses should engage in “environmentally sustainable practice” (Association of Registered Nurses of Newfoundland and Labrador, 2019, 12; CARNA, 2019, 15; College of Nurses of Ontario, 2020a, 2020b,7; College of Registered Nurses of British Columbia, 2015, 14; College of Registered Nurses of Manitoba, 2019,9; College of Registered Nurses of Prince Edward Island, 2019, 11; Nova Scotia College of Nursing, 2020, 16; Nurses Association of New Brunswick, 2020, 13; Registered Nurses Association of the Northwest Territories and Nunavut, 2019, 13; Saskatchewan Registered Nurses Association, 2019, 12).

Education was another general recommendation. The CNA (2017a, 2017b), CNA and CMA (2009), ICN (2018), NNPBC (n.d.), and RNAO (2008) recommend that nursing education, specifically undergraduate programs, incorporate environmental health topics into their programs and increase eco-literacy of nurses. In addition to education, most nursing organizations expanded their general recommendations to include systemic, practical, and research actions.

Regulatory bodies encouraged nurses to become involved in legislative and advocacy work in government and health care organizations. The NNPBC (n.d.) stated that nurses should engage in policy discussions and use their “professional voice in a unified way to advocate for the reduction in the immense footprint that health care organizations leave on the environment” (p. 3). This was echoed by the CNA (2009b, 2017a, 2017b, 2017c) and ICN (2018), who encouraged nurses to lobby governments and legislators, participate in and improve environmental policy, and advocate for the adoption of healthy public policy. CARNA (2005), RNAO (2008, 2011), and OIIQ (2019) also encouraged nurses to promote the implementation of healthy environmental policy.

Some nursing regulatory bodies and organizations detailed specific practice recommendations. These actions included reducing waste in workplace organizations by analyzing the use of single-use items (NNPBC, n.d.), and replacing disposable items with reusables (CNA & CMA, 2009). The CNA and CMA (2009) advocate for health care professionals to collaborate to form green teams, which are committees with a mandate to find and implement strategies for emphasizing environmentally healthy purchasing, training and waste programs. The CNA and CMA (2009) also encouraged nurses to take pragmatic action by educating their clients and the public about the relationship between illness and climate change, and how to incorporate healthy personal and environmental behaviors into daily activities.

Finally, many position statements identified more nursing research is required to inform health care practices that are environmentally sustainable, to enhance understanding about the impact of climate change on health, and to develop evidence to guide environmentally informed changes within the health care system and policy (CNA, 2009a, 2017b; CNA & CMA, 2009; NNPBC, n.d.). The ICN (2018) “(c)alls on governments to invest in climate change and public health research … to improve understanding of the health co-benefits of climate mitigation” (p. 3).

### Social Justice

Social justice and vulnerable populations are discussed in 19 of the 22 documents. All 11 nursing competency documents retrieved state nursing practice is inclusive of upholding social justice and supporting health in public policy (Association of Registered Nurses of Newfoundland and Labrador, 2019; CNA, 2017b; CARNA, 2019; College of Nurses of Ontario, 2020a; College of Registered Nurses of British Columbia, 2015; College of Registered Nurses of Manitoba, 2019; College of Registered Nurses of Prince Edward Island, 2019; Nova Scotia College of Nursing, 2020; Nurses Association of New Brunswick, 2020; Registered Nurses Association of the Northwest Territories and Nunavut, 2019; Saskatchewan Registered Nurses Association, 2019). Two position statements noted the First Nations and Inuit populations of Canada are at a higher risk of experiencing the effects of climate change, due to environmental impact on food sources and safe drinking water, and identified this as a growing social justice issue (CNA, 2017b; NNPBC, n.d.). TheNNPBC (n.d.), OIIQ (2019), and RNAO (2011) state that social injustice will increase due to climate change and poor environmental health, and nurses have a duty to protect vulnerable populations by engaging in environmental health advocacy. The RNAO (2011) noted environmental justice is a component of social justice and occurs when “costs of environmental damage and climate change are disproportionately borne by lower income people” (p. 3). The CNA Code of Ethics (2017) identified that nurses need to advocate for social justice, and the ICN (2018) stated “the nursing profession has a duty to contribute to climate change adaptation … and mitigation … as it is committed to protecting health and wellbeing and to promoting social justice” (p. 1). Poor environmental health exacerbates social injustices and nurses should engage in political advocacy to protect health for all (CNA, 2017a).

## Discussion

This research was conducted to explore the policies of Canadian nursing regulatory bodies and associations on nursing practice specific to environmental health. The findings of this policy analysis revealed only four of 12 nursing associations had policy statements on nursing practice and the environment. The competency statements broadly spoke of environmental sustainability and neglected to include detailed examples of environmentally sustainable practice. The misalignment between the competency documents and position statements facilitates confusion regarding whether environmental health is a priority within Canadian nursing practice. The few practical action recommendations made by the regulatory bodies may be difficult to follow due to a lack of provided information and instruction.

Nurse researchers highlighted the importance of ecoliteracy within education to ensure future nurses are aware of the health impact of the environment (MacDonnell & Buck-McFadyen, 2016; Nicholas & Breakey, 2017; NNPBC, n.d.). Environmental health should be included in nursing education through educating faculty and redeveloping the curriculum (Butterfield et al., 2014; Hewitt et al., 2006). In the United States, the Agency for Toxic Substances Disease Registry collaborated with nursing legislators and educators to develop a guide for educators to include environmental health concepts in nursing curriculums, and created academic partnerships across the country (Leffers et al., 2014). The CNA and CMA called for improved awareness and collaboration among clinicians to broaden clinical nursing skills that can be used to assess, treat and promote environmental health (2009). Florell (2020) calls for nurses to resurrect and resume health activism practices as a way to positively influence the health of the environment. Nursing education is one strategy to prepare nurses as effective health activists and to supplement nursing knowledge on environmental concerns.

Most policy statements encouraged nurses to model environmentally sustainable strategies but provided few pragmatic recommendations as to how to proceed. Nurses can increase environmental health visibility by joining groups dedicated to environmental health promotion, such as Health care without Harm (Health Care Without Harm, n.d.), the ANHE (Alliance of Nurses for Healthy Environments, n.d.) and the Canadian Association of Nurses for the Environment (Canadian Association of Nurses for the Environment, n.d.). These groups gather information and increase awareness of environmental health within practice, policy, and research. This increased visibility can lead to improved coordination within nursing and other disciplines, and increase environmental health skills, such as contributing to policy development. Advocating and becoming involved in environmentally healthy public policy was discussed in most position statements and competency documents, and nursing leaders called for nurses to become unified and politically active. To start answering the call, environmental health nursing groups need to become more visible and easily accessible for interested nurses. This can be done through providing a list of environmental health nursing groups on the websites of regulatory colleges and professional associations. This list could provide both awareness of related initiatives and a pathway for nurses to become engaged and active within environmental health groups.

Canadian nurses researchers have called for enhanced environmental health education and advocacy within nursing practice and education (Buck-McFadyen & MacDonnell, 2017). Education and policy are crucial tools to improving environmental health (Hammer et al., 2018), and all nurses would benefit from education in activism and advocacy skills development whom to contact in their local government to discuss change and how to engage in policy discussions within the workplace and the government. It is clear that environmental health is an important determinant of health, and the nursing profession can start to advocate for environmental health needs as it is a patient in dire need of care.

## Limitations

A limitation of this content analysis is the use of documents that were publicly available. The associations and colleges may have had further environmental health documents in the process of being published, or among privately held documents within the organization. As this analysis was to simulate what a practicing nurse could readily retrieve, organizations were not contacted to identify unpublished work.

## Implications

The outcome of this research has implications for research and education for nursing practice in environmental health. There is a research opportunity to identify if and how Canadian undergraduate nursing institutions are incorporating environmental health in their curriculums, and furthermore, if nursing students identify engaging in environmental sustainability as part of their scope of practice. Further research implications exist, primarily in how climate change is affecting Canadian citizens and vulnerable populations, health care economics of investing in waste reduction and clean energy, and identifying how current nurse activists and leaders engage in advocacy. As well, Canadian undergraduate and graduate nursing curriculums would benefit from a curriculum focused on environmental health, and education on political advocacy engagement to improve population health.

## Conclusions

This content analysis of Canadian nursing position statements and policies for environmental health identified limited environmental health awareness within nursing practice among competency documents and nursing position statements. Furthermore, this analysis contributed to nursing knowledge by identifying the current reduced state of environmental health focus in Canadian nursing, and current gaps within policy, education, and research. As indicated in the current competencies, nursing regulatory bodies expect nurses to include sustainable actions within their nursing practice, but do not provide relevant information and tools to successfully implement these measures. Significant opportunities currently exist for nursing leadership within environmental health nursing practice. Environmental health is a crucial determinant of human health, and nurses need to recognize the environment as a patient itself and develop relevant knowledge and skills to care for it as such.
